# Supplementary material for: Structured deep embedding model to generate composite clinical indices from electronic health records for early detection of pancreatic cancer
Source: Patterns (N Y). 2022 Dec 6;4(1):100636. doi: 10.1016/j.patter.2022.100636 (PMC9868652; doi:10.1016/j.patter.2022.100636)
Supplement: Document S1. Figures S1–S5 and Tables S1 and S3 [file mmc1.pdf]

Patterns, Volume *4*

Supplemental information

Structured deep embedding model to generate composite clinical indices from electronic health records for early detection of pancreatic cancer

Jiheum Park, Michael G. Artin, Kate E. Lee, Benjamin L. May, Michael Park, Chin Hur, and Nicholas P. Tatonetti

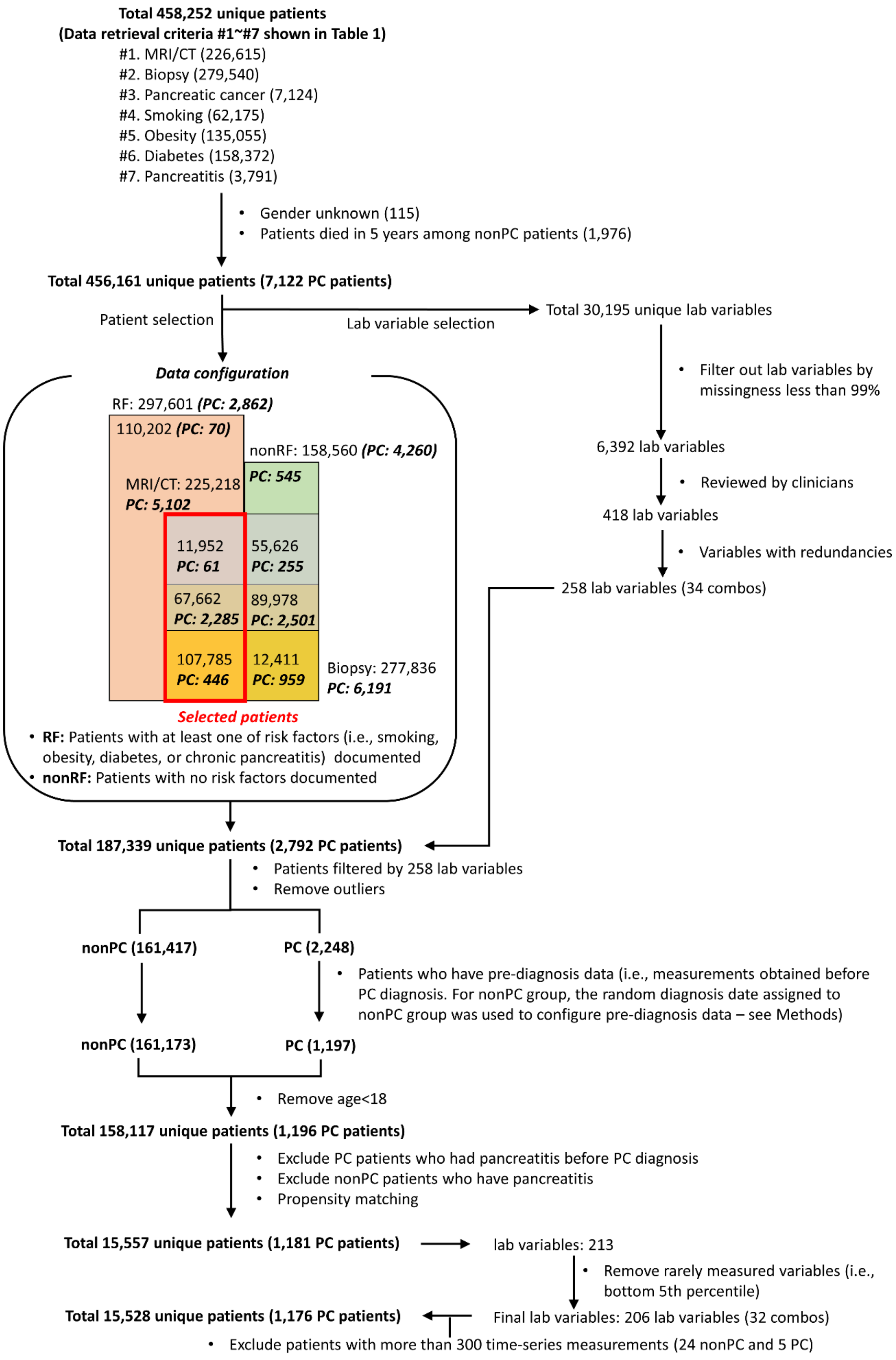
Total 458,252 unique patients
(Data retrieval criteria #1~#7 shown in Table 1)
#1. MRI/CT (226,615)
#2. Biopsy (279,540)
#3. Pancreatic cancer (7,124)
#4. Smoking (62,175)
#5. Obesity (135,055)
#6. Diabetes (158,372)
#7. Pancreatitis (3,791)
Gender unknown (115)
Patients died in 5 years among nonPC patients (1,976)
Total 456,161 unique patients (7,122 PC patients)
Patient selection
Lab variable selection
Total 30,195 unique lab variables
Filter out lab variables by missingness less than 99%
6,392 lab variables
Reviewed by clinicians
418 lab variables
Variables with redundancies
258 lab variables (34 combos)
Data configuration
RF: 297,601 (PC: 2,862)
110,202 (PC: 70)
nonRF: 158,560 (PC: 4,260)
PC: 545
MRI/CT: 225,218
PC: 5,102
11,952
PC: 61
55,626
PC: 255
67,662
PC: 2,285
89,978
PC: 2,501
107,785
PC: 446
12,411
PC: 959
Biopsy: 277,836
PC: 6,191
Selected patients
RF: Patients with at least one of risk factors (i.e., smoking, obesity, diabetes, or chronic pancreatitis) documented
nonRF: Patients with no risk factors documented
Total 187,339 unique patients (2,792 PC patients)
Patients filtered by 258 lab variables
Remove outliers
nonPC (161,417)
PC (2,248)
Patients who have pre-diagnosis data (i.e., measurements obtained before PC diagnosis. For nonPC group, the random diagnosis date assigned to nonPC group was used to configure pre-diagnosis data – see Methods)
nonPC (161,173)
PC (1,197)
Remove age<18
Total 158,117 unique patients (1,196 PC patients)
Exclude PC patients who had pancreatitis before PC diagnosis
Exclude nonPC patients who have pancreatitis
Propensity matching
Total 15,557 unique patients (1,181 PC patients)
lab variables: 213
Remove rarely measured variables (i.e., bottom 5th percentile)
Total 15,528 unique patients (1,176 PC patients)
Final lab variables: 206 lab variables (32 combos)
Exclude patients with more than 300 time-series measurements (24 nonPC and 5 PC)

Fig S1. Data preprocessing flow chart. We obtained 458,252 patient samples with 30,195 lab variables from New York-Presbyterian Hospital (NYP)/Columbia University Medical Center (CUMC) EHR data. We focused on high-risk popluation for PC (i.e., red box: selected patients), composed of the patient group who has one of the four risk factors (i.e., smoking, obesity, diabetes, or chronic pancreatitis) documented and also received either imaging or biopsy. This selected patients' data processed into the final dataset is composed of 206 lab variables in total and 15,528 patients where 1,176 are PC patients.

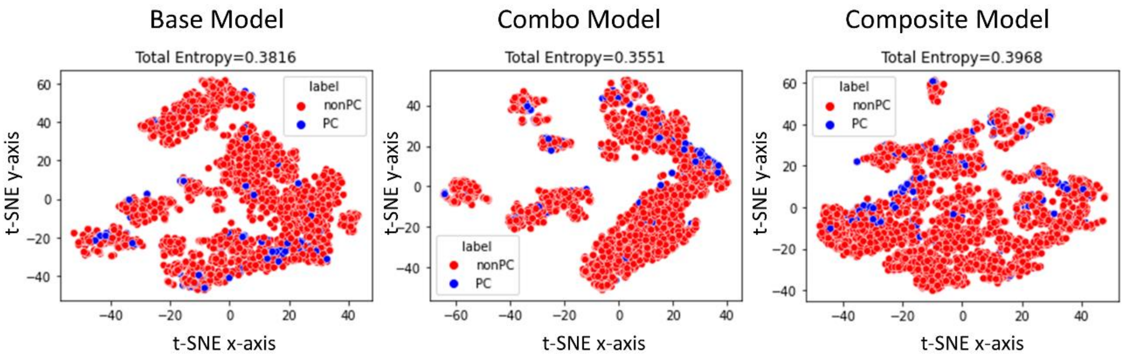

Fig S2. Cluster analysis. We evaluated clusters created by the resulting embeddings from each model (i.e., 206 embeddings from the Base Model, 32 embeddings from the Combo Model, 5 embeddings from Composite Modelg1, 3 embeddings from Composite Modelg2, and 7 embeddings from Composite Modelg3).

A Threshold>0.3

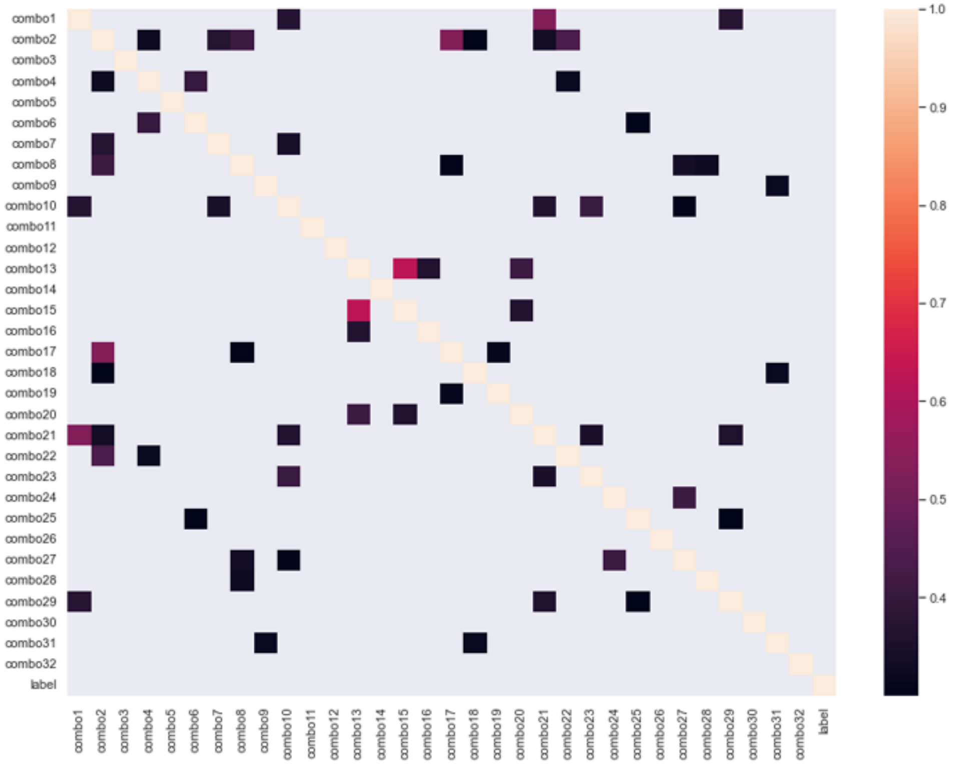

B Threshold>0.4

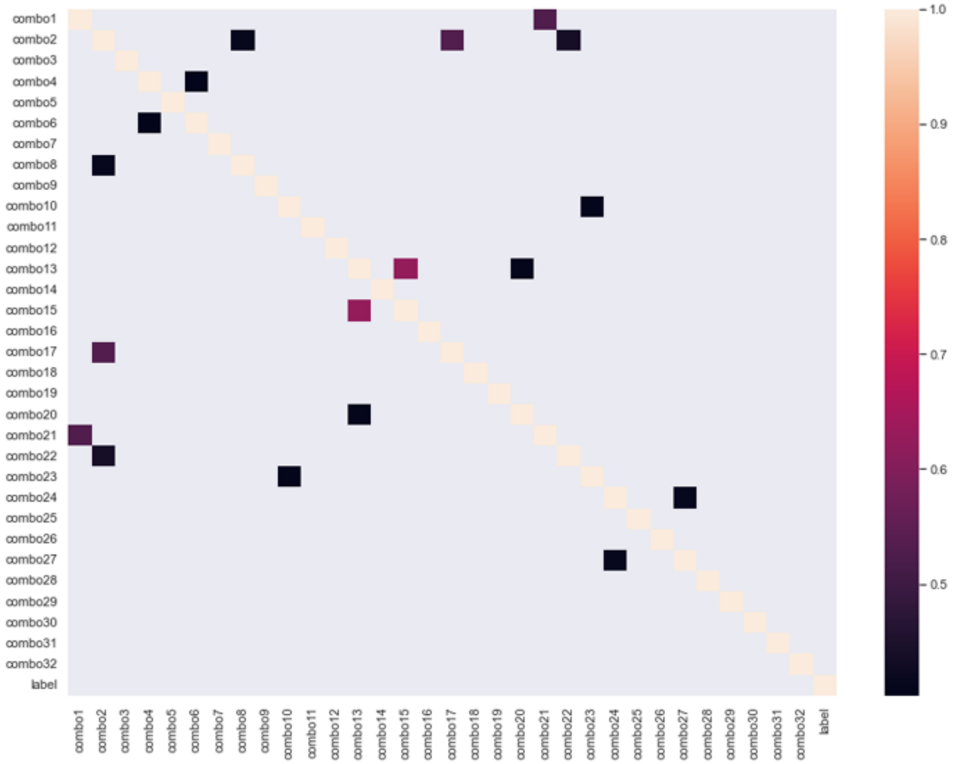

Fig S3. Correlation matrix of 32 embeddings from Combo Model. We filtered the correlation matrix by absolute value of correlation coefficients greater than 0.3 (A) and 0.4 (B) to come up with Composite $Model_{g2}$ and Composite $Model_{g3}$ respectively. We then bundled combo variables with ones that were correlated to each other (Table 1). The remaining combo variables that were not correlated with any other ones were bundled into “comp3” and “comp7” in grouping strategy 2 and 3 respectively (Table 1).

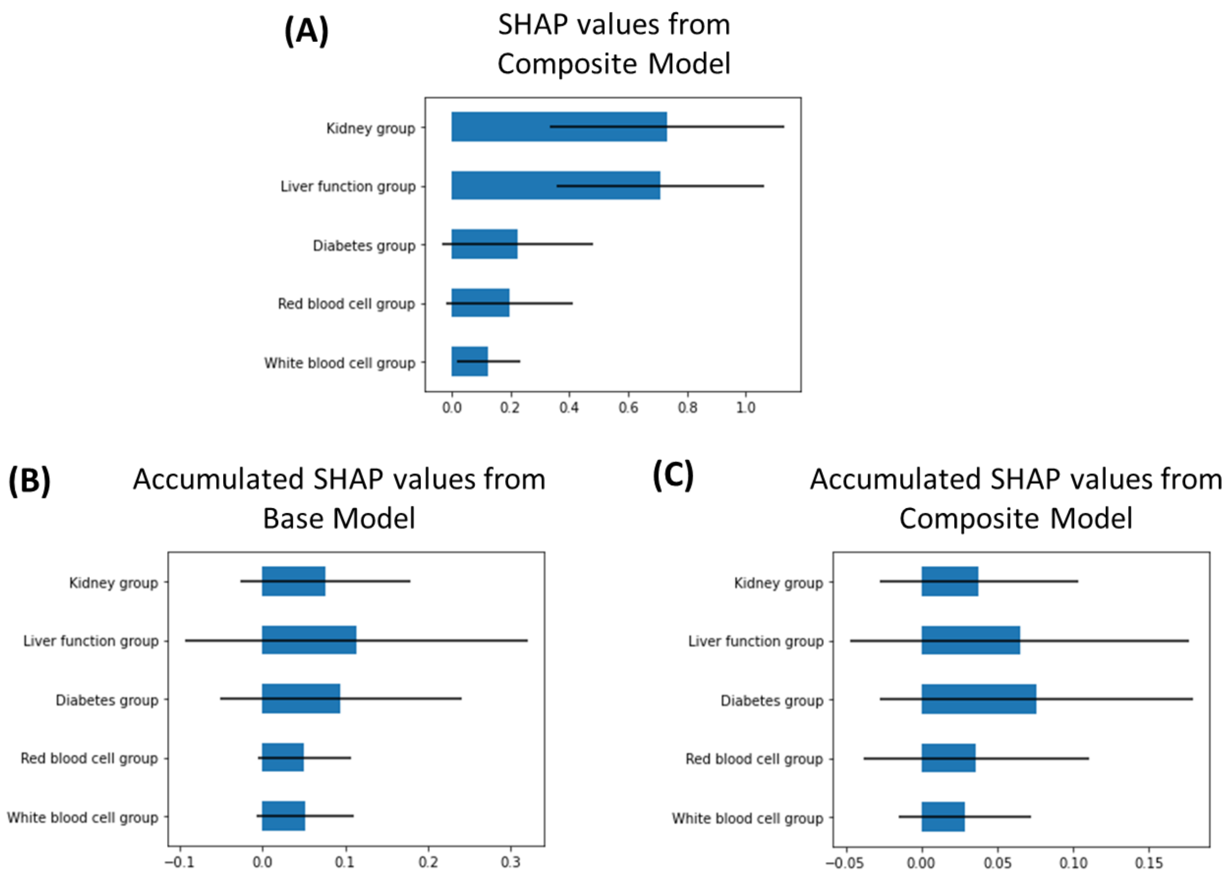

Fig S4. Accumulated feature importance test. (A) SHAP values of 5 composite indices from the Composite Model. Accumulated SHAP values of 5 composite variables from (B) the Base Model and (C) the Composite Model by grouping the SHAP values of 206 individual variables into 5 composite indices.

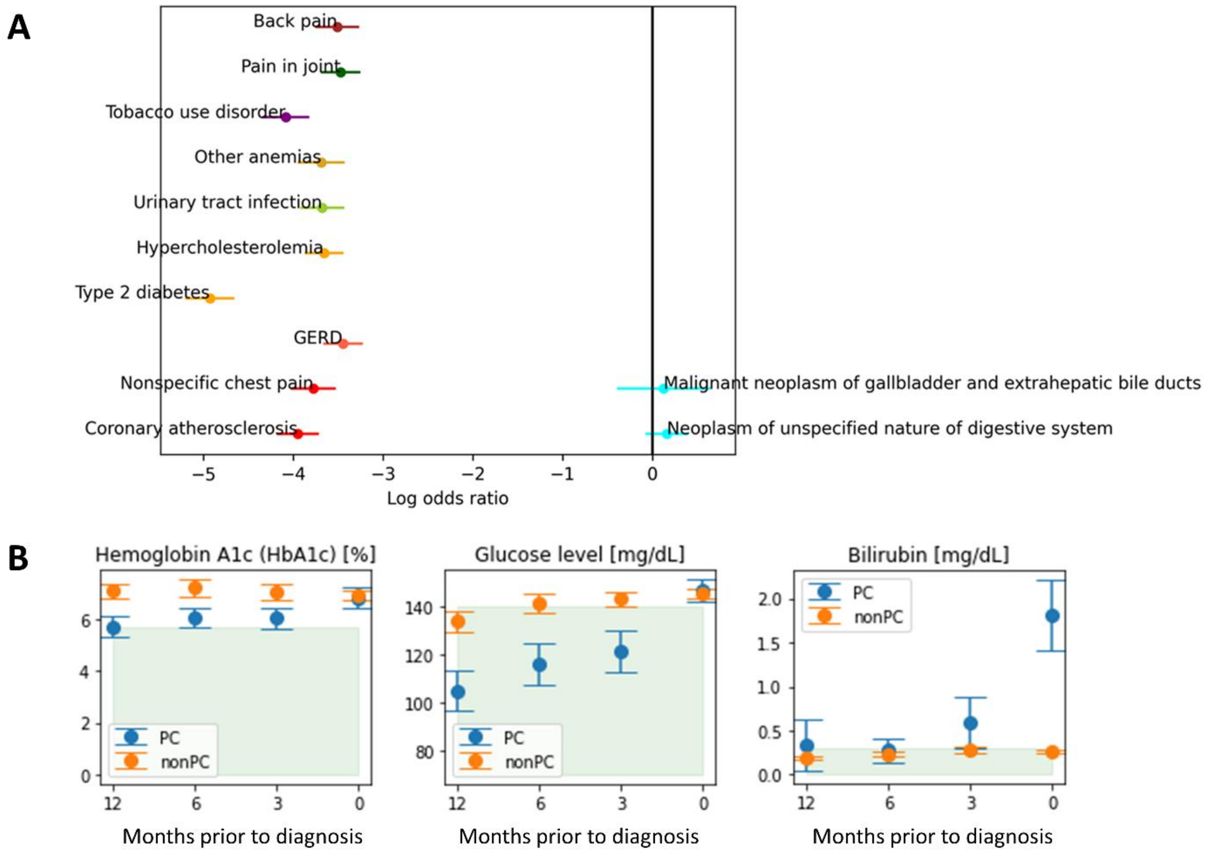

Fig S5. Phenome-wide association study (PheWAS) results. (A) Log Odds Ratio (LOR) plot where top 10 PheCodes resulted in negative LOR and all PheCodes resulted in positive LOR are shown. (B) Temporal changes in time at 0, 3, 6, and 12 months prior to diagnosis. Green shade area indicates normal ranges.

Table S1. Baseline characteristics. This table shows brief baseline characteristics for the final dataset used in the analysis. A full demographics include 7 categories of race, 8 categories of ethnicity, 66 categories of language, and 103 categories of zip codes, which are not shown in this table.

			PC/nonPC
**Total**			**1176 (8%)/14,352 (92%)**
Risk factors	Smoking	Yes	215 (18%)/2,670 (19%)
		Not documented	961 (82%)/11,682 (81%)
	Obesity	Yes	235 (20%)/2,944 (21%)
		Not documented	941 (80%)/11,408 (79%)
	Diabetes	Yes	880 (75%)/11,098 (77%)
		Not documented	296 (25%)/3,254a (23%)
Demographics	Race	White	543 (46%)/6,284 (44%)
		Asian	36 (3%)/368 (3%)
		African American	144 (12%)/1,882(13%)
		Other Combinations not described	103 (10%)/1,451 (10%)
		Unknown	344 (29%)/4,288 (30%)
	Ethnicity	Caucasian	21 (2%)/280 (2%)
		Hispanic	9 (1%)/68 (1%)
		Not Hispanic	240 (20%)/2,453 (17%)
		African American	124 (11%)/1,517 (10%)
		Unknown	778 (66%)/9,981 (70%)
	Sex	Male	631 (54%)/7,644 (53%)
		Female	545 (46%)/6,708 (47%)
	Zip code	Starts with 0 (MA, NH, ME, VT, CT, NJ)	186 (16%)/1,904 (13%)
		Starts with 1 (NY, PA)	958 (82%)/12,029 (85%)
		Starts with 3 (GA, FL, AL, TN, MS)	20 (2%)/215 (2%)

Language	English	637 (55%)/7,440 (53%)
	Spanish	103 (9%)/1,520 (11%)
	Other	311 (27%)/4,109 (29%)
	Unknown	105 (8%)/1,035 (7%)
Age		73.9 (CI95%=73.2-74.6)/74.5 (CI95%=74.3-74.7)

Table S3. Performance comparison of model results We performed 10 repetitive experiments for each model by randomly splitting the dataset into train set (80%) and test set (20%), and presented mean AUROC and AUPRC with 95% confidence intervals.

**Prediction model**	**Train set**		**Test set**	
	AUROC	AUPRC	AUROC	AUPRC
Base Model	0.873 ± 0.004	0.473 ± 0.010	0.846 ± 0.008	0.410 ± 0.020
Combo Model	0.888 ± 0.005	0.524 ± 0.017	0.855 ± 0.010	0.436 ± 0.022
Composite Model$_{g1}$	0.893 ± 0.004	0.538 ± 0.009	0.858 ± 0.009	0.435 ± 0.033
Composite Model$_{g2}$	0.893 ± 0.005	0.539 ± 0.020	0.859 ± 0.008	0.444 ± 0.025
Composite Model$_{g3}$	0.888 ± 0.006	0.523 ± 0.018	0.854 ± 0.011	0.432 ± 0.029
