# Supplementary material for: Structured deep embedding model to generate composite clinical indices from electronic health records for early detection of pancreatic cancer
Source: Patterns (N Y). 2022 Dec 6;4(1):100636. doi: 10.1016/j.patter.2022.100636 (PMC9868652; doi:10.1016/j.patter.2022.100636)
Supplement: Document S2. Article plus supplemental information [file mmc3.pdf]

Patterns

Article

Structured deep embedding model to generate composite clinical indices from electronic health records for early detection of pancreatic cancer

Highlights

- Our study shows deep-learning-based approach for generating composite indices (CIs)
- The approach incorporates the method for domain-knowledge fusion
- A strategy for grouping relevant information determines CI interpretability
- The domain-knowledge-informed CI can facilitate clinical decision-making

Authors

Jiheum Park, Michael G. Artin, Kate E. Lee, Benjamin L. May, Michael Park, Chin Hur, Nicholas P. Tatonetti

Correspondence

ch447@cumc.columbia.edu

In brief

This study presents the proof of concept for a generative deep-learning model that can distill a massive volume of EHR variables into a tractable set of composite indices that quantify the risk of cancer in a clinically meaningful and human interpretable form. With the specific aim of the early detection of pancreatic cancer, we generated five organ-specific composite indices out of 206 clinical time-series variables from EHR, and the composite index representing liver function was consistently shown to be the most important predictor.

Park et al., 2023, Patterns *4*, 100636
January 13, 2023 © 2022 The Author(s).
https://doi.org/10.1016/j.patter.2022.100636

CellPress

Patterns

CellPress
OPEN ACCESS

Article

Structured deep embedding model to generate composite clinical indices from electronic health records for early detection of pancreatic cancer

Jiheum Park,[1,9] Michael G. Artin,[2] Kate E. Lee,[3] Benjamin L. May,[4] Michael Park,[5,6] Chin Hur,[1,8,*] and Nicholas P. Tatonetti[7,8]

[1]Department of Medicine, Columbia University Irving Medical Center, New York, NY 10032, USA
[2]Hospital of the University of Pennsylvania, Philadelphia, PA 19104, USA
[3]Duke University Medical Center, Durham, NC 27710, USA
[4]Herbert Irving Comprehensive Cancer Center, Columbia University Irving Medical Center, New York, NY 10032, USA
[5]Applied Info Partners, Inc, Worlds Fair Drive, Somerset, NJ 08873, USA
[6]X-Mechanics, Cresskill, NJ 07626, USA
[7]Department of Biomedical Informatics, Columbia University, New York, NY 10032, USA
[8]Senior author
[9]Lead contact
*Correspondence: ch447@cumc.columbia.edu
https://doi.org/10.1016/j.patter.2022.100636

THE BIGGER PICTURE The explosive growth in the volume of patient medical records has resulted in an overload of information for healthcare providers, and there are currently numerous significant challenges associated with leveraging this information to improve the quality of clinical decision-making. In this work, we aim to develop new simplified representations of patient states that are both predictive and interpretable to physicians. These patient state representations are aggregated via deep-learning architectures that leverage domain knowledge to group the large number of clinical variables available per patient into a simplified set of composite indices while preserving the ability to explain how the model arrived at the final prediction. We anticipate that our methods for domain-knowledge fusion will provide a basis for producing new interpretable high-level composite indices that reduce "black box" concerns regarding model validity and therefore improve clinical adoption into decision-making.

Proof-of-Concept: Data science output has been formulated, implemented, and tested for one domain/problem

SUMMARY

The high-dimensionality, complexity, and irregularity of electronic health records (EHR) data create significant challenges for both simplified and comprehensive health assessments, prohibiting an efficient extraction of actionable insights by clinicians. If we can provide human decision-makers with a simplified set of interpretable composite indices (i.e., combining information about groups of related measures into single representative values), it will facilitate effective clinical decision-making. In this study, we built a structured deep embedding model aimed at reducing the dimensionality of the input variables by grouping related measurements as determined by domain experts (e.g., clinicians). Our results suggest that composite indices representing liver function may consistently be the most important factor in the early detection of pancreatic cancer (PC). We propose our model as a basis for leveraging deep learning toward developing composite indices from EHR for predicting health outcomes, including but not limited to various cancers, with clinically meaningful interpretations.

INTRODUCTION

Electronic health records (EHR) contain real-time, patient-centered medical records maintained by healthcare providers. EHR data offer a unique opportunity for capturing temporal variations in patients' health outcomes, which are critical for the early detection and prevention of a wide variety of health issues.[2] However, the complexity and scale of EHR data accumulates

This is an open access article under the CC BY license (http://creativecommons.org/licenses/by/4.0/).

rapidly per patient, often containing upwards of thousands of lab measurements including numerous redundancies that arise from differences in conventions across institutions, healthcare systems, and/or periodic updates.[3,4] Multiple hospital visits per patient and variations in clinical examination practices at each visit additionally contribute to this complexity.[3,5] In this situation, simplified composite indices represented by a set of grouped information could significantly reduce the volume of data required to facilitate more informed clinical decision-making.

Standard deep neural network architectures are often formulated as black-box models, as input variables are combined in an untraceable manner to produce uninterpretable predictions.[6] In this study, we designed a structured deep embedding model that preserves variable relationships by confining the mixing of variables to respect groupings that were determined by domain experts (e.g., clinicians). By incorporating this clinician-designed grouping strategy into the design of the neural network architecture, we increase transparency in the deep-learning process, thus improving interpretability. The lack of such transparency has been a major drawback in the field of healthcare, where unexplainable errors or biases can inform incorrect clinical decisions.[7–10] We hypothesized that the incorporation of a domain knowledge informed grouping strategies for such high-dimensional inputs may constrain deep-learning models from fitting spurious correlations and result in improved model performance as well as interpretability of the results. The intermediate model outputs may also be suggestive of potential variable groupings that result in composite indices of particular clinical utility.

To investigate our hypothesis, we used longitudinal laboratory test results in EHR data from the New York-Presbyterian (NYP) Columbia University Irving Medical Center (CUIMC) data warehouse. We focused on patients at high risk for pancreatic cancer (PC), the same cohort used in our previous study.[1] Due to the limited understanding of risk factors associated with the early presentation of PC, PC is mostly found in late stages with regional spread (29%) and distant metastasis (52%).[11,12] Although there are several known risk factors associated with PC, such as family history, genetic syndromes, and chronic diseases,[13] currently no clear screening or surveillance guidelines exist to identify and screen high-risk populations. The addition of new pre-screening variables could therefore significantly improve risk prediction to the point where targeted screening and surveillance may be effective.

Current efforts in the field of artificial intelligence (AI) related to PC have primarily focused on imaging data. The use of AI-based methodology is particularly lacking with respect to clinical data from the EHR. Risk modeling based on longitudinal data with cutting-edge AI techniques has been emphasized as one of the future directions to actively explore that may enhance the early detection of PC.[14] Recently, Placido et al. applied AI to trajectories of International Classification of Diseases (ICD) codes of 6 million patients, where 24,000 were diagnosed with PC to predict PC risk. The author tested various models including multilayer perceptron (MLP), transformers, and gated recurrent units (GRUs). The best performance model achieved an area under the receiver operating characteristic curve (AUROC) of 0.88 for cancer occurrence within 36 months using transformers. The AUROC from cross-application of the model on an external dataset, however, decreased to 0.78, which addresses the limitation of model generalizability, likely due to different coding practices across different health systems.[15] The Med-BERT, a contextualized embedding model pre-trained on a structured EHR dataset of 28,490,650 patients, has shown some promise for establishing a generalizable AI model for medical/clinical applications. Med-BERT enables utilization of small local training datasets for realistic disease prediction tasks.[16] Another study presented by Rasmy et al. evaluated effects of data granularity on prediction performance. For example, the study compared prediction performance between the model using the diagnosis information as originally recorded (i.e., ICD codes) and the grouped information such as phenome-wide association studies (PheWASs) which groups raw ICD codes into 1,820 categories. More specifically, they emphasize that the grouped information resulted in surprisingly good performance compared with other terminologies with higher levels of granularity (e.g., Unified Medical Language System [UMLS]). They further pointed out that grouping strategies are practically useful due to the improvements in human readability that arise from effective reductions in the dimensionality of data.[17]

In this study, we developed our model with laboratory measurement data, in contrast to most models based on EHR data that rely primarily on ICD codes. Although the generalizability of our results remains unconfirmed due to limitations in our sample sizes and sources, we have focused on the evaluation of our conceptual framework of creating composite indices using AI.

To this end, we demonstrate a protocol for incorporating expert-domain information into deep-learning architectures with the aim of combining redundant information toward the generation of composite indices (i.e., combining information on individual measures into a single representative value). By examining different grouping strategies and evaluating their clinical predictive utility, our approach provides a basis for the use of deep learning for the development of clinically interpretable indices that can measure and predict health outcomes.

RESULTS

We present three structured deep embedding models that are ordered by the level of hierarchy in the variable groupings according to a taxonomy developed by domain experts (Figure 1): (1) a base model composed of 1st hierarchical grouping where time-series data of each variable (i.e., 206 in total) are individually embedded into one-dimensional embeddings; (2) a combo model, where a 2nd hierarchical grouping is added to merge redundant variables into one-dimensional embeddings and create 32 combo embeddings; and (3) a composite model, where a 3rd hierarchical grouping is added in which combo variables are further grouped according to their relevance (e.g., organ systems) determined by domain experts into one-dimensional embeddings, which we named composite indices. For the composite model, we examined three different ways of defining the relevance among combo variables (Table 1: grouping strategy 1, grouping strategy 2, and grouping strategy 3); see experimental procedures for more details. We then systematically evaluated the grouping effects on overall prediction performance and examined predictivity of the resultant composite indices by measuring feature importance and cluster qualities. The detailed pre-processing of the data, baseline characteristics of the final dataset, and the list of lab

CellPress
OPEN ACCESS

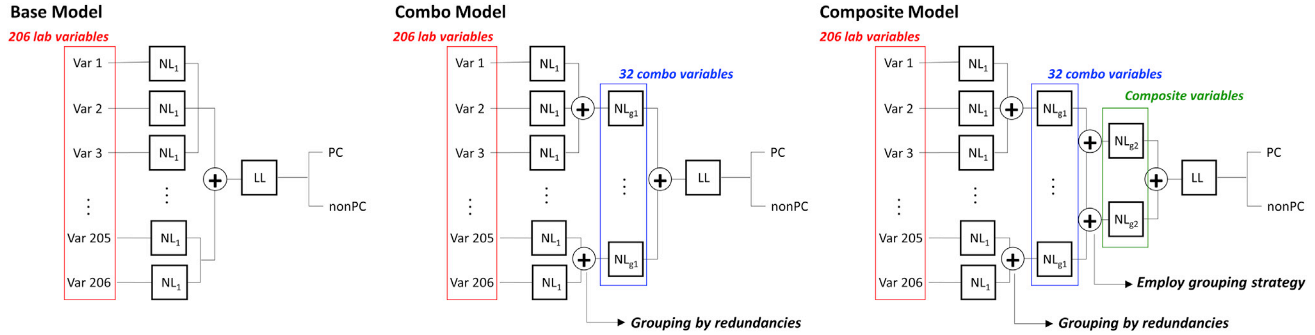

Figure 1. Structured deep embedding models

We designed three different structured deep embedding models with different levels of hierarchy: (1) base model: 1st level hierarchy, (2) combo model: 2nd level hierarchy, and (3) composite model: 3rd level hierarchy. We set output dimension of neural networks from each embedding layer to be 1. Thus, the number of embeddings from the final layer of each model are 206, 32, and 5 (in case of grouping strategy 1), respectively. We tested three grouping strategies (Table 1) for creating composite indices, one based on organ systems guided by clinicians' input (composite model$_{g1}$) and two others (composite model$_{g2}$ and composite model$_{g3}$) based on correlation among the resultant 32 combo embeddings (Figure S3) from the combo model.

variables used in this analysis are shown in the supplemental information (Figure S1; Tables S1 and S2, respectively).

The structured deep embedding model has no adverse effects on the model performance

All three types of structured deep embedding model (Figure 1) presented similar prediction performance (Figure 2A; Table S3). The prediction performances for early detection slightly varied depending on the grouping design (Figure 2B) but were comparable overall. We evaluated clusters created by the resulting embeddings from each model (i.e., 206 embeddings from the base model, 32 embeddings from the combo model, 5 embeddings from composite model$_{g1,}$ 3 embeddings from composite model$_{g2}$, and 7 embeddings from composite model$_{g3}$). The total entropy of those clusters from each model presented negligible differences (Figure 2C). The t-stochastic neighbor embedding (t-SNE) method was not able to show clear clusters within any of the embeddings (Figure S2), indicating that PC and non-PC are not easily separable.

The structured grouping strategy improves model interpretability when the grouping patterns are clinically interpretable by domain experts

We performed feature importance evaluations on composite indices from composite model$_{g1}$, composite model$_{g2}$, and composite model$_{g3}$, respectively, using importance scores assigned by logistic regression, decision tree, random forest, and xgboost. While composite indices from composite model$_{g1}$ were represented by particular organ systems determined by domain experts, those from composite model$_{g2}$ and composite model$_{g3}$ were inspired by learned representations from deep-learning algorithms (i.e., correlations between 32 combo embeddings resulted from the combo model; Figure S3). Unlike grouping strategy 1, which categorizes clinical variables according to the organ systems, grouping strategy 2 and grouping strategy 3 resulted in mixtures of those variables with no clinically discernable patterns (Table 1).

For composite model$_{g1}$, the feature importance measures from decision tree, random forest, and xgboost, which offer importance scores based on the reduction in the criterion used to select split points, consistently showed that the liver function group and the kidney function group have relatively higher importance associated with PC prediction. The importance measures from logistic regression showed slightly different results but commonly showed the liver function group to have relatively high importance. For composite model$_{g2}$, all four algorithms indicated that composite indice #1 (comp1) is the most important composite index, whereas for composite model$_{g3}$, the results suggested that the importance of all seven composite indices are comparable (Figure 3).

Shapely additive explanation (SHAP) analysis on the structured deep embedding model reveals interactions of individual predictors for making a final prediction

To understand interactions among grouping layers in composite model$_{g1}$, we used the SHAP method,[18] which measures contributions of each component (i.e., SHAP interaction values) to the final prediction (Figure 4) using a game-theoretic approach. Among the five composite indices (i.e., white blood cell group, red blood cell group, liver function group, kidney group, and diabetes group), the kidney group and the liver function group were inferred to have relatively high importance for predicting PC, which is consistent with the results from tree-based algorithms in section (the structured grouping strategy improves model interpretability when the grouping patterns are clinically interpretable by domain experts). At the level of 1st hierarchical grouping, alkaline phosphatase was ranked as the most important variable, though it was not statistically significant with respect to the subsequent 14 variables, thus suggesting a comparable contribution from the first 15. We highlighted those top 15 individual variables and their affiliated combo variables, which were also sorted according to their SHAP values, within the same color to examine the connections between the 1st hierarchical grouping layer and the 2nd hierarchical grouping layer. From this, we observe that the top 15 variables were mostly associated with the combo variables that were also placed at the top. In connection with the 3rd hierarchical grouping layer, 10 out of those 15 variables were associated with either the kidney group or the liver function group. The box frame of combo variable names has been colored

Table 1. Three grouping strategies tested with composite model

Grouping strategy 1		Grouping strategy 2		Grouping strategy 3	
Composite indices	Combo variables	Composite indices	Combo variables	Composite indices	Combo variables
White blood cell group	1 PCT neutrophils	comp1	1 PCT neutrophils	comp1	1 PCT neutrophils
	5 PCT basophils		2 ALT		21 creatinine
	19 WBC		4 AST	comp2	2 ALT COMBO
	22 PCT eosinophils		6 bilirubin direct		22 PCT eosinophils
	25 PCT monocytes		7 bilirubin direct		17 phosphorus
	29 PCT lymphocytes		8 ALK PHOS		8 ALK PHOS
	31 ABS neutrophils		9 calciu	comp3	4 AST
	32 ABS basophils		10 PT		6 bilirubin direct
Red blood cell group	11 RBC		18 platelets	comp4	10 PT
	12 RDW		17 phosphorus		23 glucose
	18 platelets		19 WBC	comp5	13 sodium
	26 hemoglobulin		21 creatinine		20 chloride
	27 MCH		22 PCT eosinophils		15 potassium
	28 hematocrit		23 glucose	comp6	24 MCV
	24 MCV		24 MCV		27 MCH
Liver function group	2 ALT		25 PCT monocytes	comp7	3 serum albumin
	3 serum albumin		27 MCH		5 PCT basophils
	4 AST		28 hematocrit		7 bilirubin indirect
	6 bilirubin direct		29 PCT lymphocytes		9 calcium
	7 bilirubin indirect		31 ABS neutrophils		11 RBC
	8 ALK PHOS	comp2	13 sodium		12 RDW
	10 PT		20 chloride		16 total protein
	16 total protein		15 potassium		14 HBA1C
Kidney group	9 calcium		16 total protein		18 platelets
	13 sodium	comp3	3 serum albumin		19 WBC
	15 potassium		5 PCT basophils		
	17 phosphorus		11 RBC		
	20 chloride		12 RDW		
	30 magnesium		14 HBA1C		
	21 creatinine		26 hemoglobin		
Diabetes group	14 HBA1C		30 magnesium		
	23 glucose		32 ABS basophils		

The 32 combo embeddings from combo model were grouped according to their organ system (grouping strategy 1) and their correlation matrix (grouping strategy 2 and grouping strategy 3). Please see Table S2 for the individual variables that comprise each combo variable. The numbers in front of each combo variable from 1 to 32 correspond to the numbers shown in Table S2. PCT, percent [%]; PT, prothrombin time; ALK, alkaline; PHOS, phosphorus; ABS, absolute; RBC, red blood cell; RDW, red cell diameter width; WBC, white blood cell; MCV, mean cell volume; MCH, mean cell hemoglobin.

according to the color frame of composite indices to observe interactions between the 2nd and the 3rd hierarchical grouping layers. This visualization shows that the top 50% combo variables were mostly associated with the kidney group (yellow) and the liver function group (green), while the bottom 50% were mostly with the red blood cell group (blue) and the white blood cell group (red).

In addition to the above analysis incorporating all pre-diagnosis data, we conducted the same analysis with the dataset composed of measurements obtained at least 12 months prior to diagnosis. In this case, the diabetes group presented the highest SHAP value followed by the kidney group and the liver function group. Consistently, the glucose combo variable was shown as the top contributor. Compared with the analysis incorporating all pre-diagnosis data, the SHAP analysis at 12 months prior to diagnosis resulted in reduced SHAP values overall (Figure 4).

We also evaluated the accumulated SHAP values of 5 composite variables from the base model and the composite model by grouping the SHAP values of 206 individual variables into 5 composite indices. These results were different from theSHAP values of 5 composite indices resulted from the composite model (Figure S4), which demonstrates that the composite model is not a redundant model with the base model and that the SHAP values are dependent on the model architecture.

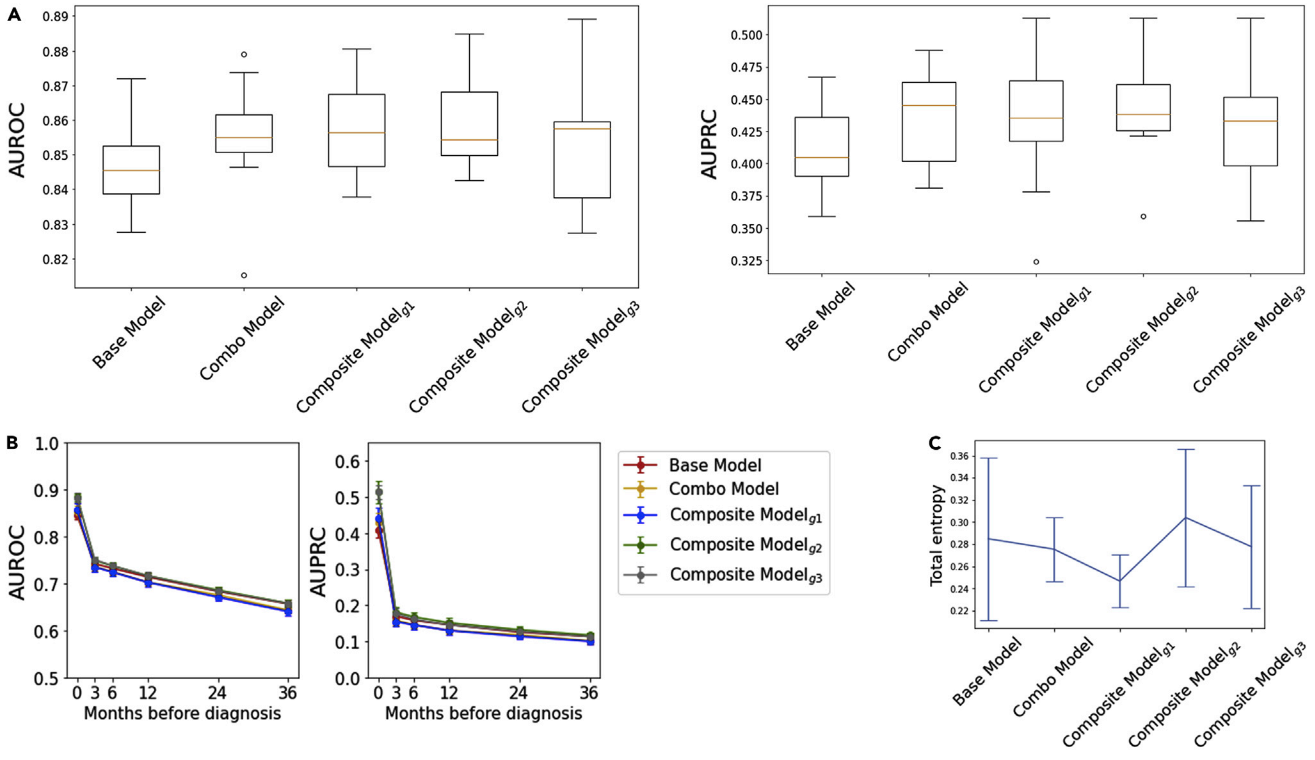

Figure 2. The structured deep embedding model has no adverse effects on the model performance

(A) The prediction performance scores for all five models were similar although slightly improved with the structured deep embedding model that has more hierarchical groupings (e.g., base model versus composite model$_{g1}$). The boxplots show the "minimum," 1st quartile (Q1, 25th percentile), median (Q2, 50th percentile), and 3rd quartile (Q3, 75th percentile), and the "maximum," where the minimum and maximum values are defined as Q1 – 1.5 * interquartile range (IQR) and Q3 + 1.5 * IQR, respectively.

(B) Early detection performance by measuring predictivity with the limited dataset available at the months prior to diagnosis. The error bars indicate 95% confidence intervals.

(C) We measured total entropy of clusters (i.e., purity of the clusters classified into PC and non-PC) resulted from each model. The error bars indicate 95% confidence intervals.

DISCUSSION

A large and increasing volume of clinical information poses significant challenges for effective clinical decision-making. Motivated by the success of deep-learning applications for information distillation such as text summarization, we built a structured deep embedding model that leverages domain-specific taxonomies to generate a simplified set of composite indices containing the most relevant predictive information from a massive collection of input variables.

The impact of our structured deep-learning architecture on model performance and interpretability is influenced by several considerations. On one hand, the grouping strategy leverages domain knowledge from human experts to constrain the training process of the neural network, thereby eliminating potentially spurious correlations that can result in overfitting. Additionally, the flow of information through the network is forced to follow explainable pathways, thus enabling a clear visualization of how the model arrives at the final prediction (Figure 4). Conversely, these restrictions could also prevent the network from exploring all possible pathways, particularly those that may be unintuitive to clinicians, which could negatively impact performance. However, our study results indicate that the use of structured grouping patterns that are clinically interpretable by domain experts results in improvements in model interpretability without any adverse impact on model predictions (Figure 2). For example, in this study, we systematically evaluated various levels of hierarchical groupings, a base model with the 1st level groupings, a combo model with the 2nd level groupings, and a composite model with the 3rd level groupings. All three types of structured deep embedding models (Figure 1) presented similar prediction performance. (Figure 2A; Table S3). Furthermore, we tested three distinct grouping strategies (Table 1) within the composite model framework: the first one designed based on clinical domain knowledge of organ system classifications (i.e., grouping strategy 1), and two others based on correlation matrices of 32 combo embeddings that result from the combo model (i.e., grouping strategies 2 and 3; Figure S3). While grouping strategy 1 represents "expert curation," grouping strategies 2 and 3 represent random groupings based on "deep-learning curation." All three grouping strategies showed negligible differences in prediction performance between each other as well as compared with the base model and the combo model, which demonstrates that the grouping strategy does not negatively impact model performance. On the other hand, the grouping strategy was found to significantly

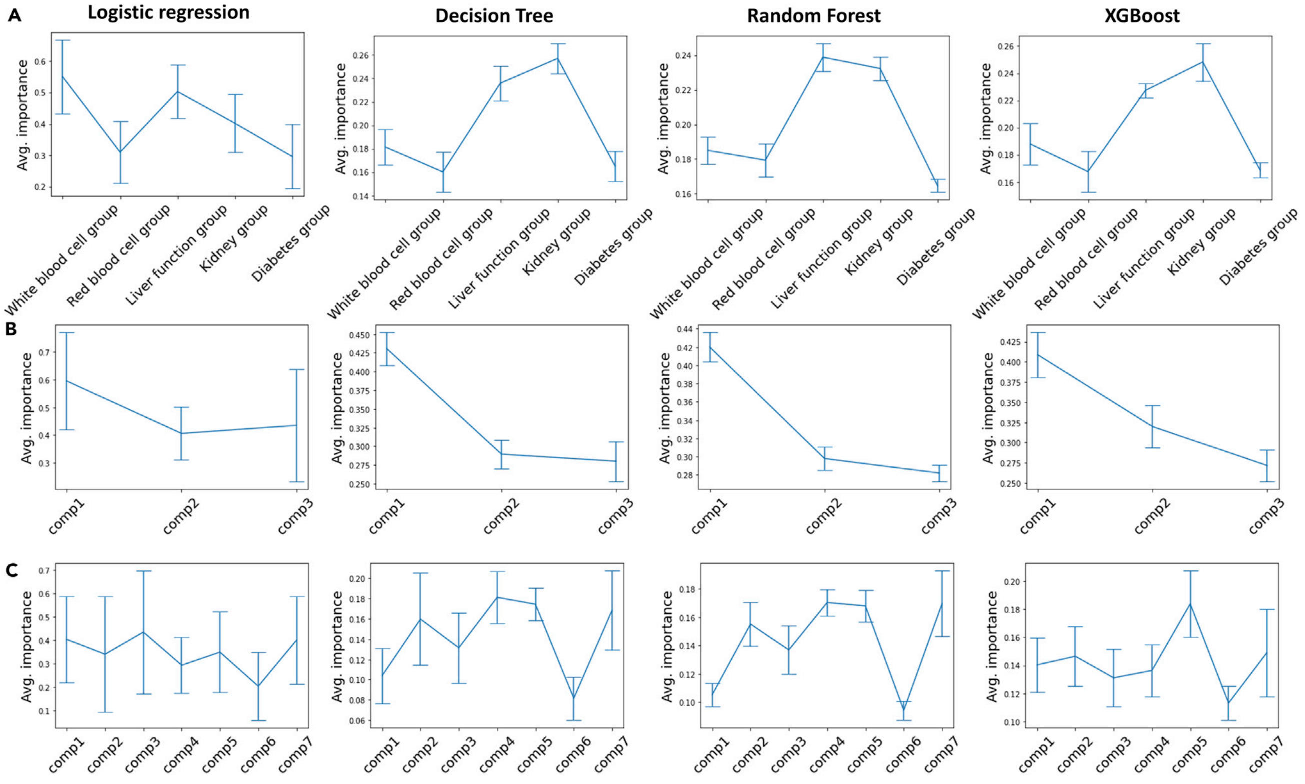

Figure 3. The structured grouping strategy improves model interpretability when the grouping patterns are clinically interpretable by domain experts

(A–C) We used four algorithms to measure feature importance of composite indices: logistic regression, decision tree, random forest, and xgboost. The feature importance was evaluated on five composite embeddings from (A) the composite model$_{g1}$, where the importance measures from logistic regression showed slightly different results but commonly showed that the liver function group is relatively more important, (B) the composite model$_{g2}$, where all four algorithms indicated that comp1 is relatively more important than others, and (C) the composite model$_{g3}$, where importance of all seven composite indices is comparable. The error bars indicate standard deviations.

improve model interpretability when implemented with human-interpretable structures as in grouping strategy 1. Strategies based on statistical similarity metrics such as grouping strategy 2 and grouping strategy 3 resulted in more obscure patterns that were difficult to deconstruct from a clinical perspective (Table 1), thus limiting their explainability. For example, comp1 from grouping strategy 2, which generated a composite index with the greatest contribution to the final prediction (Figure 3), was not found to provide clinically meaningful insights.

To further demonstrate interpretability of the structured deep embedding model, we applied a SHAP analysis to composite model$_{g1}$ and evaluated the interactions between the grouped layers by measuring the SHAP values of each component. To investigate correlations specific to early warning signs of PC, we also conducted the SHAP analysis on a dataset containing only measurements that were obtained at least 12 months prior to PC diagnosis. The analysis using all pre-diagnosis data showed that the kidney function group and the liver function group were consistently the most important predictors for the early detection of PC, which provides intriguing support for a growing body of clinical evidence supporting this observation. For example, both chronic and acute kidney failure are known as potential complications of acute pancreatitis,[19–21] where recent studies have revealed that acute pancreatitis may be an early symptom of PC.[22–24] Liver function tests are also a common consideration when diagnosing PC. For example, bilirubin measurements, which are indicators of liver function, are expected to be elevated in patients with PC, either because the tumor blocks the bile ducts, leading to a buildup of bilirubin in the blood stream, or because the PC has metastasized to the liver.[25] Thus, we may observe a bilirubin increase in the time leading up to the diagnosis. However, at 12 months prior to diagnosis, we observed increased importance of the diabetes group as an early predictor, although it was not significant compared with either the composite indices of the kidney group or the liver function group.

Limitations of our study include a lack of conclusive evaluation of its generalizability, which may be affected by the selection of the non-PC control group from subpopulations associated with significant pre-existing medical comorbidities relative to the general population (Figure S1). For example, one of the most distinctive differences observed between the PC and non-PC groups at 12 months prior to diagnosis was their serum glucose levels. However, the PheWAS results showed that type 2 diabetes had the greatest negative log odds ratio (LOR = −4.91, Figure S5A), thus suggesting that the relatively high serum glucose levels observed in the non-PC group would more likely be explained by a difference in the progression of diabetes in this population. Using ICD codes and their given dates, we confirmed

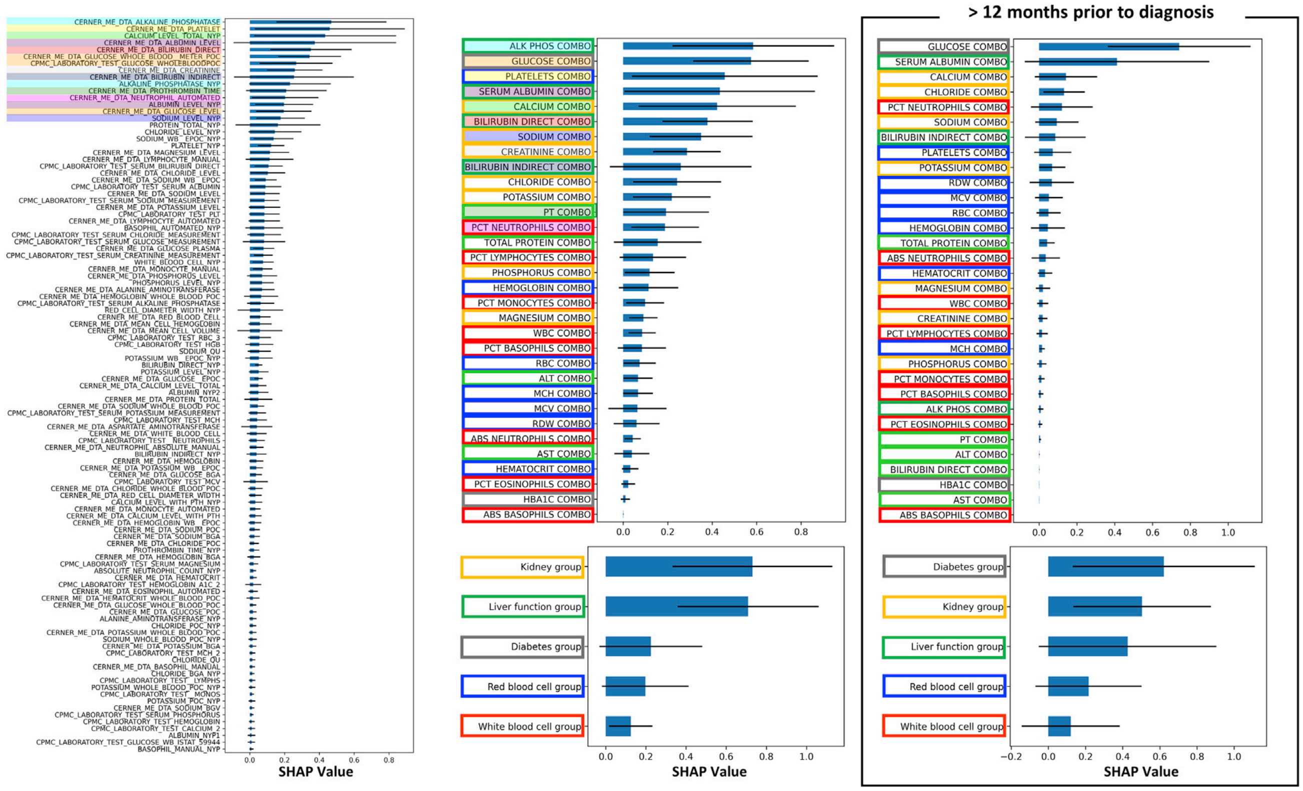

Figure 4. Shapely additive explanation (SHAP) analysis on the structured deep embedding model reveals interactions of individual predictors for making a final prediction

We measured SHAP values to understand interactions among grouping layers in composite modelg_1. We highlighted the top 15 individual variables from the 1st hierarchical grouping layer and their affiliated combo variables in the 2nd hierarchical grouping layer within the same color. We then color framed the combo variable names according to the color frame of composite indices in the 3rd hierarchical grouping layer to examine connections between layers. The error bars indicate standard deviations.

that many patients with PC received diabetes diagnoses after they were diagnosed with PC, while many non-PC patients were consistently diagnosed with diabetes in earlier months. Despite this, it is notable that the glucose levels in the PC group gradually increased as serial measurements approached the date of PC diagnosis (Figure S5B), which is consistent with observations of clinical phenomena in the literature, suggesting that new-onset diabetes could be a potential early indicator of PC.[26] Considering the importance of temporal variations in health outcomes for understanding disease occurrence, the incorporation of sequence-based neural networks such as recurrent neural network (RNN) and autoregressive transformers[27] may provide more insightful results.

Our goal is to establish a detailed pattern of trends in lab measurements that could alert clinicians to give extra attention to affected patients with respect to PC risk, though a lack of generalizability limits the application our results in practice. Building upon our demonstration of composite indices as new risk factors and their risk scores (SHAP values) for PC, future work could focus on applying the attention mechanism[28] in the model to produce risk scores of composite indices for individual patients. A byproduct of its achievement would be the generation of representative composite indices analogous to body mass index (BMI; a composite index of height and weight), which currently has high clinical utility in the diagnosis and risk assessment of numerous adverse health conditions.[29] Unsupervised machine-learning frameworks such as autoencoding[29] architectures, which are trained to efficiently compress and reconstruct the input data, would likely improve generalizability, particularly when dealing with big data composed of massive patient records spanning multiple sources. For example, Le at al. theoretically and empirically demonstrated that a neural network that jointly predicts targets and inputs (reconstruction) improves generalization compared with the standard neural network.[30] Another future direction would be an expansion to additional EHR modalities. For example, the groupings based on patients' hospital visits (i.e., 1st visit, 2nd visit, etc.) could be used to further improve prediction accuracy, as has been demonstrated in previous studies.[31] In addition, different genetic alterations may be associated with different risk factors, and incorporating these well-motivated data sources into the design of embedding structure may further refine our assessment of risk factors.[32]

To implement our proposed concept of using composite indices in a real clinical setting, we will expand cohorts used to training the model to any patients with gastrointestinal (GI) appointments and demonstrate clinical utility of the composite indices by running subcohorts with different endpoints related

to GI diseases including stenosis, hemorrhoids, colon cancer, liver cancer, and stomach cancer. Upon successfully establishing the model architecture generating validated composite indices, we will be able to develop a user interface (UI) displaying a few numbers of composite indices instead of thousands of raw EHR data. This UI system will allow clinicians to obtain overall insights of individual patients on his/her health conditions and to access more detailed information by clicking composite indices of interest, which will lead to displaying the higher level of variables consisting of those composite indices and their individual contributions to risk prediction.

EXPERIMENTAL PROCEDURES

Resource availability

Lead contact

Further information and requests for resources should be directed to the lead contact, Jiheum Park (jp4147@cumc.columbia.edu).

Materials availability

This study did not generate any new unique materials.

Data and code availability

The raw EHR data reported in this study cannot be deposited in a public repository due to Health Information Portability and Accountability Act (HIPPA) regulations. Details on the raw EHR data pre-processing are provided in the supplemental information as well as our previous work.[1] The code used in the development of the structured deep embedding models and the analysis has been deposited at Zenodo under https://doi.org/10.5281/zenodo.7232859 and is publicly available as of the date of publication.

Data collection and preparation

We used the NYP/CUIMC EHR dataset from a previous publication that was curated for conducting early detection of PC.[1] The detailed flow chart depicting the data processing pathway is shown in Figure S1.

For lab variables, we eliminated ones containing missing values for more than 99% of patients, resulting in 6,392 unique variables, from which 418 of the most clinically relevant variables were identified by domain experts based on common standards[33]. Among the 418 variables, we identified 258 variables with redundancies (i.e., reported in different lab names but essentially the same measurements). After further data pre-processing, including the configuration of pre-diagnosis data and the propensity score matching, the number of the final set of variables was reduced to 206. Grouping by redundant variables, we created 32 bundled variables from those 206 individual variables, and we call them combo variables (Table S2).

Configuring pre-diagnosis data

We configured the PC dataset into pre-diagnosis data by eliminating the lab measurements obtained after or at the time of their first PC diagnosis date. Based on the average percentage reduction of the total number of measurements in this process of removing post-diagnosis data and configuring the data into pre-diagnosis data for each variable, we assigned random diagnosis dates for non-PC patients and configured the non-PC dataset into pre-diagnosis data. A more detailed description can be seen in our previous study.[1] We substituted missing values with 0.

Propensity score matching

In order to eliminate confounding biases in lab measurements due to baseline characteristics (e.g., race, ethnicity, sex, zip code, patient language, age, smoking, obesity, diabetes), our final negative control group was selected on the basis of matching the full joint probability distributions of these observables. This was done systematically with propensity score matching using the Pymatch package for Python (v.3.9). We performed 100 iterations of fits to the logistic regression model, given the imbalance of the data (i.e., 158,117 non-PC versus 1,196 PC; Figure S1), and measured average accuracy, stopping at an accuracy close to 50% (implying inseparability of the two populations in the data). Through the propensity score matching procedure, we reduced the separability resulting from the baseline characteristics from 72% to 55%.

Structured deep embedding models with grouping strategies

We designed three different structured deep embedding models with different levels of hierarchy: (1) base model: 1st level hierarchy, (2) combo model: 2nd level hierarchy, and (3) composite model: 3rd level hierarchy (Figure 1). The base model consists of two sequential components: an embedding layer followed by a prediction layer. In the embedding layer, an independent set of trainable weights were used to learn a dimensionally reduced representation of each time-series variable, thus producing one simplified feature vector for each sequence of measurements. These learned feature vectors were then concatenated and passed through the prediction layer, which uses a simple linear transformation to project the data to a binary prediction space using the standard log softmax function. The combo model has a grouping layer added after an embedding layer that groups redundant variables identified in Table S2 and generates 32 combo embeddings (i.e., combos). The composite model has another grouping layer added after the layer that groups redundant variables to group relevant variables among 32 combos according to the grouping strategies and to create composite indices. Since our goal is to evaluate utility of one representative value (e.g., composite index), we set the output dimension of neural networks from each embedding layer to be 1.

We tested three grouping strategies for creating composite indices, one based on organ systems guided by clinicians' input (composite model$_{g1}$) and two others (composite model$_{g2}$ and composite model$_{g3}$) based on the correlation among the resultant 32 combo variables (Figure S3; Table 1) from the combo model. We used the correlation matrix filtered by the values greater than 0.3 for composite model$_{g2}$ and 0.4 for composite model$_{g3}$ (Figure S3), followed by bundling combo variables by the ones that are correlated to each other. The remaining combo variables that were not correlated with any other ones were bundled into comp3 in composite model$_{g2}$ and comp7 in composite model$_{g3}$ respectively (Table 1). The higher threshold, for example >0.5, resulted in very few numbers of variables in correlation.

Model training

We performed 10 repetitive experiments for each model by randomly splitting the dataset into a train set (80%) and a test set (20%). For each experiment, we used early stopping in a 50 epoch training loop by monitoring loss on the test set. We used AUROC and area under the precision-recall curve (AUPRC) as performance evaluation metrics.

For evaluating early prediction performance, we created PC datasets composed of earlier times on the basis of the date when patients received a PC diagnosis code. For example, to provide model prediction at 12 months prior to diagnosis, we tested the model trained with pre-diagnosis data with a dataset containing lab results that were measured more than 12 months prior to diagnosis.

Data analysis

Clustering

Using the resultant composite indices (i.e., one-dimensional embeddings) from the model, we performed clustering analysis to quantify their classification performance. We used the t-SNE[34] method for clustering and plotted, in a two-dimensional (2D) map, where each datapoint is colored in accordance with PC and non-PC (Figure S2). To calculate the entropy of PC and non-PC datapoints, we used Gaussian mixture model (GMM)[35] clustering initialized with K-means[36] for subgroup labeling. The total entropy was measured by following the equation[37]

$$\sum_i \left(\sum_j (n_{ij} / n_i) \log(n_{ij} / n_i) \times w_{ij} \right),$$

where n_{ij} indicates the number of datapoints labeled in j in cluster i and w_{ij} indicates the relative weight of class label j in cluster i.

Feature importance

We tested four different classification methods on composite embeddings from composite model$_{g1}$, composite model$_{g2}$, and composite model$_{g3}$, respectively, to calculate feature importance scores and highlight the most relevant composite indices to the target (e.g., PC): (1) logistic regression, (2) decision tree,[38] (3) random forest,[38] and (4) xgboost.[39]

Model interpretability

To provide further interpretations of the model, we used SHAP, which identifies and visualizes important interactions made in the model. SHAP estimates the impact of each feature on the prediction for every observation (e.g., patient), while algorithms such as tree-based methods or logistic regression produce a single ranking of all features. We presented the mean absolute SHAP value of each feature over all patients for every grouping layer involved in composite model$_{g1}$.

PheWAS

We analyzed the ICD signature of patients with PC compared with the control group by performing a binary PheWAS to identify comorbidities associated with each patient group. We used the pyPheWAS toolkit, an open-source Python package[40]

First, we identified all ICD-9 and ICD-10 codes that patients have received along the hospital visits. Second, ICD codes were mapped to corresponding PheCodes, which includes 1,866 hierarchical phenotype codes, and sorted according to 18 general categories (Figure S5A). Since the ICD mapping in the package does not cover the full range of ICD-9 and ICD-10 codes, 34% of ICD codes were removed in PheWAS. We found that many ICD-10 codes in our dataset were not included in the ICD mapping. In order to minimize those removal rate, we converted ICD-10 codes that do not exist in the mapping to corresponding ICD-9 using the web scraping technique[41]. We used Beautiful Soup, the most widely used Python library for web scraping, for parsing HTML from https://www.icd10data.com/Convert and converting ICD-10 to ICD-9, or vice versa, to see if the converted codes exist in the mapping. As a result, the removal rate dropped to 20%. Finally, we performed mass logistic regression across all PheCodes using *pyPhewasModel* in the toolkit.

Temporal analysis

To further investigate individual lab components involved in composite indices (i.e., white blood cell group, red blood cell group, liver function group, kidney group, diabetes group), we examined temporal changes in time at 0, 3, 6, and 12 months prior to diagnosis (Figure S5B). At each month of the measurement, we used mean values of the measurements that were recorded within 2 months before and after from that particular month for each patient.

In order to conduct quantitative assessment on temporal changes in the measurements, we applied linear regression and measured the coefficients, which represent slope of the fitted lines. Considering that the normal ranges for each variable are different, the slope also needed to be adjusted according to those normal ranges. We measured adjusted slope (i.e., coefficient$_{adjusted}$) by dividing the resultant coefficient by the diagonal line slope of the normal range window (i.e., green shade area shown in Figure S5B).

Statistics

We presented the results in either mean values with 95% confidence intervals (CIs 95) or standard deviations, or boxplots with the "minimum," 1st quartile (Q1, 25th percentile), median (Q2, 50th percentile), and 3rd quartile (Q3, 75th percentile), and the "maximum," where the minimum and maximum values are defined as Q1–1.5 * interquartile range (IQR) and Q3 + 1.5 * IQR, respectively. A two-sample t test was used in the comparative analysis. A p value of less than 0.05 was considered statistically significant.

SUPPLEMENTAL INFORMATION

Supplemental information can be found online at https://doi.org/10.1016/j.patter.2022.100636.

ACKNOWLEDGMENTS

This work was supported by National Cancer Institute of the National Institutes of Health under 1R21CA265400-01.

AUTHOR CONTRIBUTIONS

Data curation, B.L.M. and J.P.; data selection and review, J.P., K.E.L., M.G.A., and C.H.; conceptualization, J.P., M.P., C.H., and N.P.T.; methodology, J.P., M.P., C.H., and N.P.T.; formal analysis, J.P., M.P., and M.G.A.; investigation, J.P., M.P., C.H., and N.P.T.; supervision, M.P., C.H., and N.P.T.; writing – original draft, J.P., M.G.A., K.E.L., M.P., C.H., and N.P.T.; writing – review & editing, J.P., K.E.L., M.P., C.H., and N.P.T.

DECLARATION OF INTERESTS

The authors declare no competing interests.

Received: July 5, 2022
Revised: August 18, 2022
Accepted: October 24, 2022
Published: December 6, 2022

REFERENCES

1. Park, J., Artin, M.G., Lee, K.E., Pumpalova, Y.S., Ingram, M.A., May, B.L., Park, M., Hur, C., and Tatonetti, N.P. (2022). Deep learning on time series laboratory test results from electronic health records for early detection of pancreatic cancer. J. Biomed. Inform. *131*, 104095. https://doi.org/10.1016/j.jbi.2022.104095.
2. Ehrenstein, V., Kharrazi, H., Lehmann, H., and Taylor, C.O. (2019). Obtaining data from electronic health records. In Tools and Technologies for Registry Interoperability, Registries for Evaluating Patient Outcomes: A User's Guide, Third Edition, Richard E. Gliklich, et al., eds. (Agency for Healthcare Research and Quality (US)). Addendum 2 [Internet].
3. Crew, D., and Houser, S.H. (2021). Overcoming challenges of merging multiple patient Identification and matching systems: a case study. Perspect. Health Inf. Manag. *18*, 1n.
4. Kim, E., Rubinstein, S.M., Nead, K.T., Wojcieszynski, A.P., Gabriel, P.E., and Warner, J.L. (2019). The Evolving Use of Electronic Health Records (EHR) for Research*4* (Elsevier), pp. 354–361.
5. Tayefi, M., Ngo, P., Chomutare, T., Dalianis, H., Salvi, E., Budrionis, A., and Godtliebsen, F. (2021). Challenges and opportunities beyond structured data in analysis of electronic health records. WIREs Comp. Stat. *13*, e1549.
6. Petch, J., Di, S., and Nelson, W. (2022). Opening the black box: the promise and limitations of explainable machine learning in cardiology. Can. J. Cardiol. *38*, 204–213. https://doi.org/10.1016/j.cjca.2021.09.004.
7. Zhao, J., Wang, T., Yatskar, M., Ordonez, V., and Chang, K.-W. (2017). Men also like shopping: reducing gender bias amplification using corpus-level constraints. Preprint at arXiv. https://doi.org/10.48550/arXiv.1707.09457.
8. Gilpin, L.H., Bau, D., Yuan, B.Z., Bajwa, A., Specter, M., and Kagal, L. (2018). Explaining Explanations: An Overview of Interpretability of Machine Learning (IEEE), pp. 80–89.
9. Nguyen, A., Yosinski, J., and Clune, J. (2015). Deep Neural Networks Are Easily Fooled: High Confidence Predictions for Unrecognizable Images (In Proceedings of the IEEE conference on computer vision and pattern recognition), pp. 427–436.
10. Ledford, H. (2019). Millions of black people affected by racial bias in health-care algorithms. Nature *574*, 608–609.
11. Wagner, M., Redaelli, C., Lietz, M., Seiler, C.A., Friess, H., and Büchler, M.W. (2004). Curative resection is the single most important factor determining outcome in patients with pancreatic adenocarcinoma. Br. J. Surg. *91*, 586–594. https://doi.org/10.1002/bjs.4484.
12. Seigel, R., and Jemal, A. (2015). American Cancer Society: Cancer Facts and Figures 2015 (American Cancer Society).
13. Rawla, P., Sunkara, T., and Gaduputi, V. (2019). Epidemiology of pancreatic cancer: global trends, etiology and risk factors. World J. Oncol. *10*, 10–27. https://doi.org/10.14740/wjon1166.
14. Kenner, B.J., Abrams, N.D., Chari, S.T., Field, B.F., Goldberg, A.E., Hoos, W.A., Klimstra, D.S., Rothschild, L.J., Srivastava, S., Young, M.R., and Go, V.L.W. (2021). Early detection of pancreatic cancer: applying artificial intelligence to electronic health records. Pancreas *50*, 916–922. https://doi.org/10.1097/MPA.0000000000001882.
15. Placido, D., Yuan, B., Hjaltelin, J.X., Haue, A.D., Chmura, P.J., Yuan, C., Kim, J., Umeton, R., Antell, G., and Chowdhury, A. (2022). Pancreatic cancer risk predicted from disease trajectories using deep learning. Preprint at bioRxiv. https://doi.org/10.1101/2021.06.27.449937.

16. Rasmy, L., Xiang, Y., Xie, Z., Tao, C., and Zhi, D. (2021). Med-BERT: pretrained contextualized embeddings on large-scale structured electronic health records for disease prediction. NPJ Digit. Med. *4*, 1–13.

17. Rasmy, L., Tiryaki, F., Zhou, Y., Xiang, Y., Tao, C., Xu, H., and Zhi, D. (2020). Representation of EHR data for predictive modeling: a comparison between UMLS and other terminologies. J. Am. Med. Inform. Assoc. *27*, 1593–1599.

18. Lundberg, S.M., and Lee, S.I. (2017). A unified approach to interpreting model predictions. Adv Neur *30*, 4768–4777.

19. Nassar, T.I., and Qunibi, W.Y. (2019). AKI associated with acute pancreatitis. Clin. J. Am. Soc. Nephrol. *14*, 1106–1115. https://doi.org/10.2215/CJN.13191118.

20. Golay, V., and Roychowdhary, A. (2012). Acute pancreatitis in chronic kidney disease–a common but often misunderstood combination. Ren. Fail. *34*, 1338–1340. https://doi.org/10.3109/0886022X.2012.718951.

21. Kroner, P.T., Mareth, K., Raimondo, M., Lee, D.D., Alsaad, A., Aslam, N., Abader, P., and Wadei, H.M. (2019). Acute pancreatitis in advanced chronic kidney disease and kidney transplant recipients: results of a US nationwide analysis. Mayo Clin. Proc. Innov. Qual. Outcomes *3*, 160–168. https://doi.org/10.1016/j.mayocpiqo.2019.03.006.

22. Liu, J., Wang, Y., and Yu, Y. (2020). Meta-analysis reveals an association between acute pancreatitis and the risk of pancreatic cancer. World J. Clin. Cases *8*, 4416–4430. https://doi.org/10.12998/wjcc.v8.i19.4416.

23. Rijkers, A.P., Bakker, O.J., Ahmed Ali, U., Hagenaars, J.C.J.P., van Santvoort, H.C., Besselink, M.G., Bollen, T.L., and van Eijck, C.H.; Dutch Pancreatitis Study (2017). risk of pancreatic cancer after a primary episode of acute pancreatitis. Pancreas *46*, 1018–1022. https://doi.org/10.1097/MPA.0000000000000879.

24. Zhang, X., An, R., Tian, H., and Zhao, J. (2019). Increased risk of pancreatic cancer after acute pancreatitis: a meta-analysis of prospective cohort studies. Clin. Res. Hepatol. Gastroenterol. *43*, e39–e41. https://doi.org/10.1016/j.clinre.2018.09.008.

25. Haas, M., Heinemann, V., Kullmann, F., Laubender, R.P., Klose, C., Bruns, C.J., Holdenrieder, S., Modest, D.P., Schulz, C., and Boeck, S. (2013). Prognostic value of CA 19-9, CEA, CRP, LDH and bilirubin levels in locally advanced and metastatic pancreatic cancer: results from a multicenter, pooled analysis of patients receiving palliative chemotherapy. J. Cancer Res. Clin. Oncol. *139*, 681–689.

26. Pannala, R., Basu, A., Petersen, G.M., and Chari, S.T. (2009). New-onset diabetes: a potential clue to the early diagnosis of pancreatic cancer. Lancet Oncol. *10*, 88–95. https://doi.org/10.1016/S1470-2045(08)70337-1.

27. Katharopoulos, A., Vyas, A., Pappas, N., and Fleuret, F. (2020). Transformers Are Rnns: Fast Autoregressive Transformers with Linear Attention (PMLR), pp. 5156–5165.

28. Niu, Z., Zhong, G., and Yu, H. (2021). A review on the attention mechanism of deep learning. Neurocomputing *452*, 48–62.

29. Tschannen, M., Bachem, O., and Lucic, M. (2018). Recent Advances in Autoencoder-Based Representation Learning. Preprint at arXiv. https://doi.org/10.48550/arXiv.1812.05069.

30. Le, L., Patterson, A., and White, M. (2018). Supervised autoencoders: improving generalization performance with unsupervised regularizers. Adv. Neural Inf. Process. Syst. *31*.

31. Choi, E., Xiao, C., Stewart, W.F., and Sun, J.M. (2018). MiME: multilevel medical embedding of electronic health records for predictive healthcare. Adv. Neural Inf. Process. Syst. *31*, 31.

32. Saif, M.W., Karapanagiotou, L., and Syrigos, K. (2007). Genetic alterations in pancreatic cancer. World J. Gastroenterol. *13*, 4423–4430.

33. Luo, Y., Szolovits, P., Dighe, A.S., and Baron, J.M. (2016). Using machine learning to predict laboratory test results. Am. J. Clin. Pathol. *145*, 778–788.

34. Cieslak, M.C., Castelfranco, A.M., Roncalli, V., Lenz, P.H., and Hartline, D.K. (2020). t-Distributed stochastic neighbor embedding (t-SNE): a tool for eco-physiological transcriptomic analysis. Mar. Genomics *51*, 100723. https://doi.org/10.1016/j.margen.2019.100723.

35. Bouman, C.A., Shapiro, M., Cook, G., Atkins, C.B., and Cheng, H. (1997). Cluster: An Unsupervised Algorithm for Modeling Gaussian Mixtures (The Board of Trustees of Purdue University).

36. Tzortzis, G.F., and Likas, A.C. (2009). The global kernel k-means algorithm for clustering in feature space. IEEE Trans. Neural Netw. *20*, 1181–1194. https://doi.org/10.1109/TNN.2009.2019722.

37. Sripada, S.C., and Rao, M.S. (2011). Comparison of purity and entropy of k-means clustering and fuzzy c means clustering. Indian journal of computer science and engineering *2*.

38. Rakhra, M., Soniya, P., Tanwar, D., Singh, P., Bordoloi, D., Agarwal, P., Takkar, S., Jairath, K., and Verma, N. (2021). Crop price prediction using random forest and decision tree regression:-A review. Mater. Today Proc.

39. Chen, T., and Guestrin, C. (2016). Xgboost: A Scalable Tree Boosting System (In Proceedings of the 22nd acm sigkdd international conference on knowledge discovery and data mining), pp. 785–794.

40. Kerley, C.I., Chaganti, S., Nguyen, T.Q., Bermudez, C., Cutting, L.E., Beason-Held, L.L., Lasko, T., and Landman, B.A. (2022). pyPheWAS: A Phenome-Disease Association Tool for Electronic Medical Record Analysis. Neuroinformatics *20*, 1–23.

41. Saurkar, A.V., Pathare, K.G., and Gode, S.A. (2018). An overview on web scraping techniques and tools. Int. J. Future Revol.comput. Sci.Commun. Eng. *4*, 363–367.

Patterns, Volume *4*

Supplemental information

Structured deep embedding model to generate composite clinical indices from electronic health records for early detection of pancreatic cancer

Jiheum Park, Michael G. Artin, Kate E. Lee, Benjamin L. May, Michael Park, Chin Hur, and Nicholas P. Tatonetti

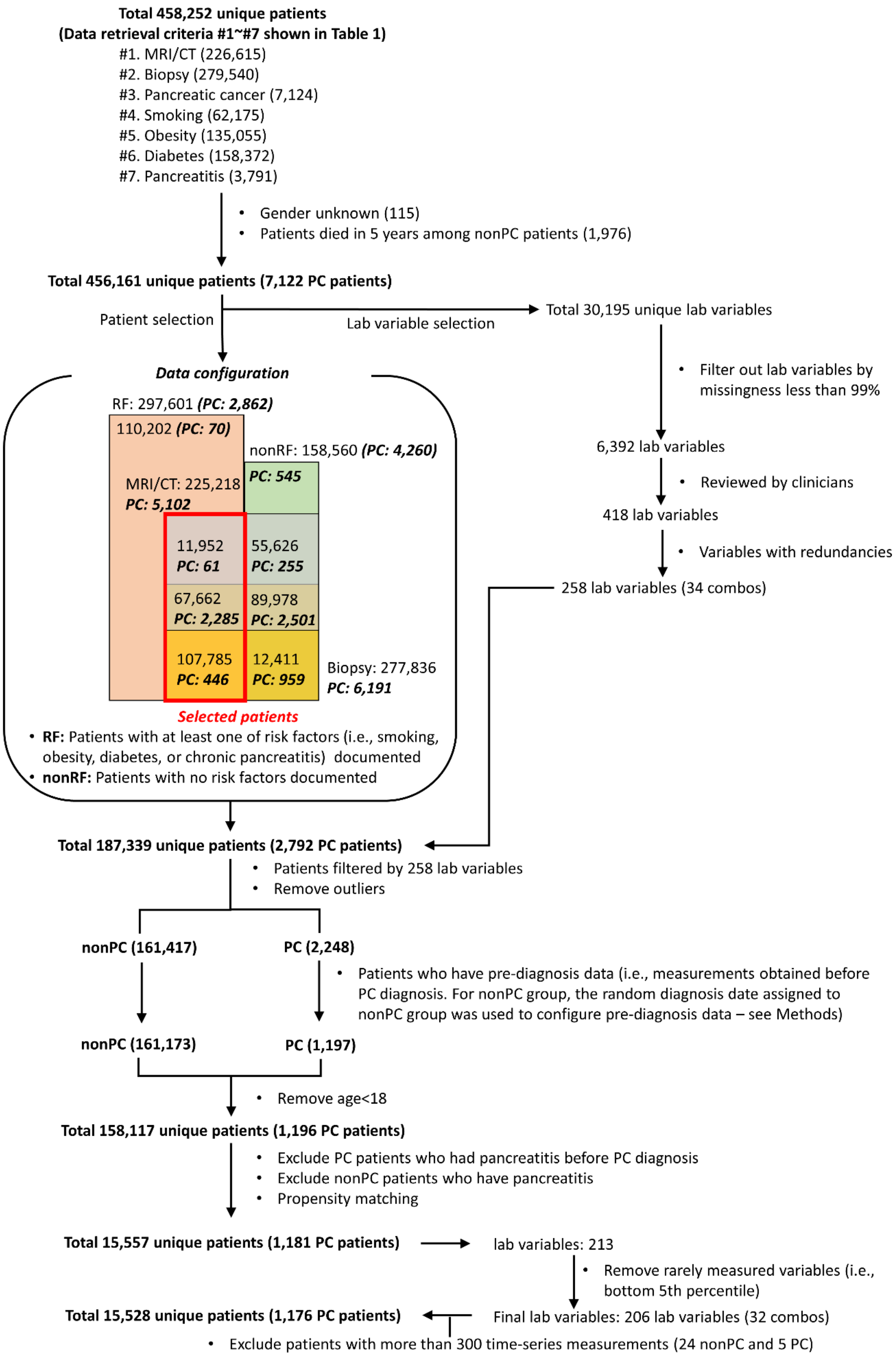
Total 458,252 unique patients
(Data retrieval criteria #1~#7 shown in Table 1)
#1. MRI/CT (226,615)
#2. Biopsy (279,540)
#3. Pancreatic cancer (7,124)
#4. Smoking (62,175)
#5. Obesity (135,055)
#6. Diabetes (158,372)
#7. Pancreatitis (3,791)
• Gender unknown (115)
• Patients died in 5 years among nonPC patients (1,976)
Total 456,161 unique patients (7,122 PC patients)
Patient selection
Lab variable selection
Total 30,195 unique lab variables
• Filter out lab variables by missingness less than 99%
6,392 lab variables
• Reviewed by clinicians
418 lab variables
• Variables with redundancies
258 lab variables (34 combos)
Data configuration
RF: 297,601 (PC: 2,862)
110,202 (PC: 70)
nonRF: 158,560 (PC: 4,260)
PC: 545
MRI/CT: 225,218
PC: 5,102
11,952
PC: 61
55,626
PC: 255
67,662
PC: 2,285
89,978
PC: 2,501
107,785
PC: 446
12,411
PC: 959
Biopsy: 277,836
PC: 6,191
Selected patients
• RF: Patients with at least one of risk factors (i.e., smoking, obesity, diabetes, or chronic pancreatitis) documented
• nonRF: Patients with no risk factors documented
Total 187,339 unique patients (2,792 PC patients)
• Patients filtered by 258 lab variables
• Remove outliers
nonPC (161,417)
PC (2,248)
• Patients who have pre-diagnosis data (i.e., measurements obtained before PC diagnosis. For nonPC group, the random diagnosis date assigned to nonPC group was used to configure pre-diagnosis data – see Methods)
nonPC (161,173)
PC (1,197)
• Remove age<18
Total 158,117 unique patients (1,196 PC patients)
• Exclude PC patients who had pancreatitis before PC diagnosis
• Exclude nonPC patients who have pancreatitis
• Propensity matching
Total 15,557 unique patients (1,181 PC patients)
lab variables: 213
• Remove rarely measured variables (i.e., bottom 5th percentile)
Total 15,528 unique patients (1,176 PC patients)
Final lab variables: 206 lab variables (32 combos)
• Exclude patients with more than 300 time-series measurements (24 nonPC and 5 PC)

Fig S1. Data preprocessing flow chart. We obtained 458,252 patient samples with 30,195 lab variables from New York-Presbyterian Hospital (NYP)/Columbia University Medical Center (CUMC) EHR data. We focused on high-risk popluation for PC (i.e., red box: selected patients), composed of the patient group who has one of the four risk factors (i.e., smoking, obesity, diabetes, or chronic pancreatitis) documented and also received either imaging or biopsy. This selected patients' data processed into the final dataset is composed of 206 lab variables in total and 15,528 patients where 1,176 are PC patients.

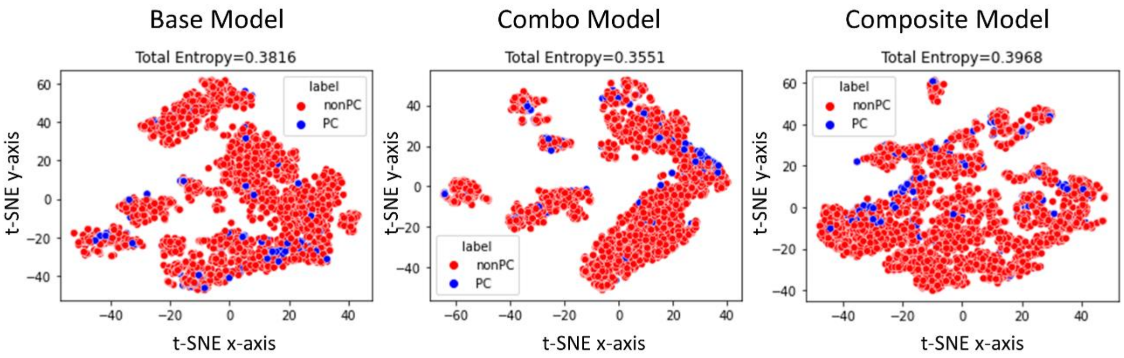

Fig S2. Cluster analysis. We evaluated clusters created by the resulting embeddings from each model (i.e., 206 embeddings from the Base Model, 32 embeddings from the Combo Model, 5 embeddings from Composite Modelg1, 3 embeddings from Composite Modelg2, and 7 embeddings from Composite Modelg3).

A Threshold>0.3

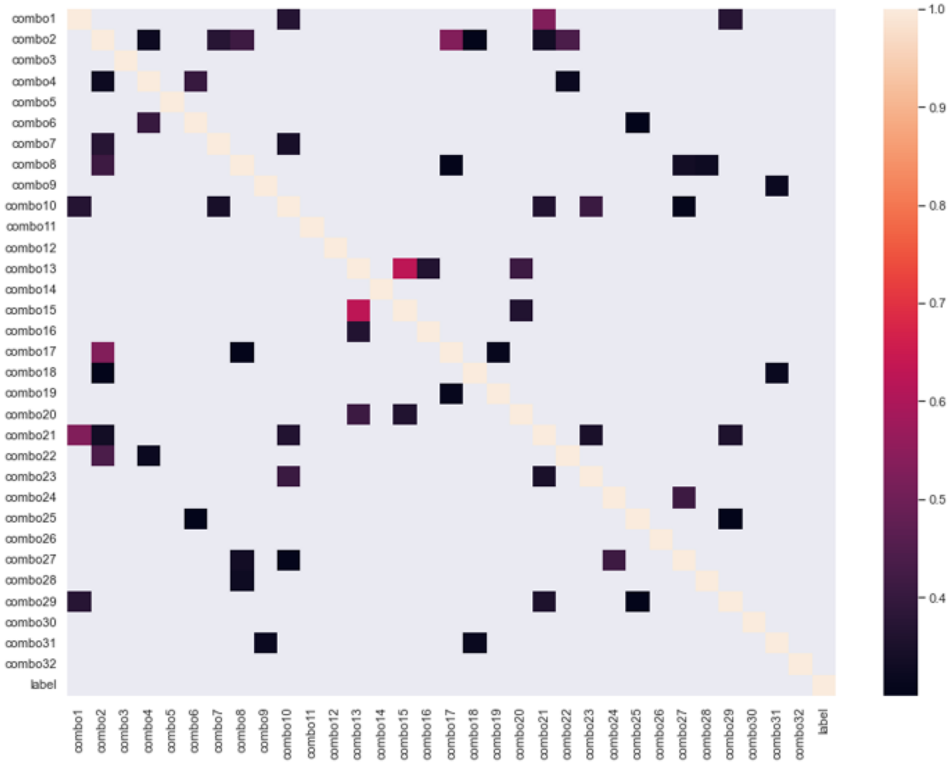

B Threshold>0.4

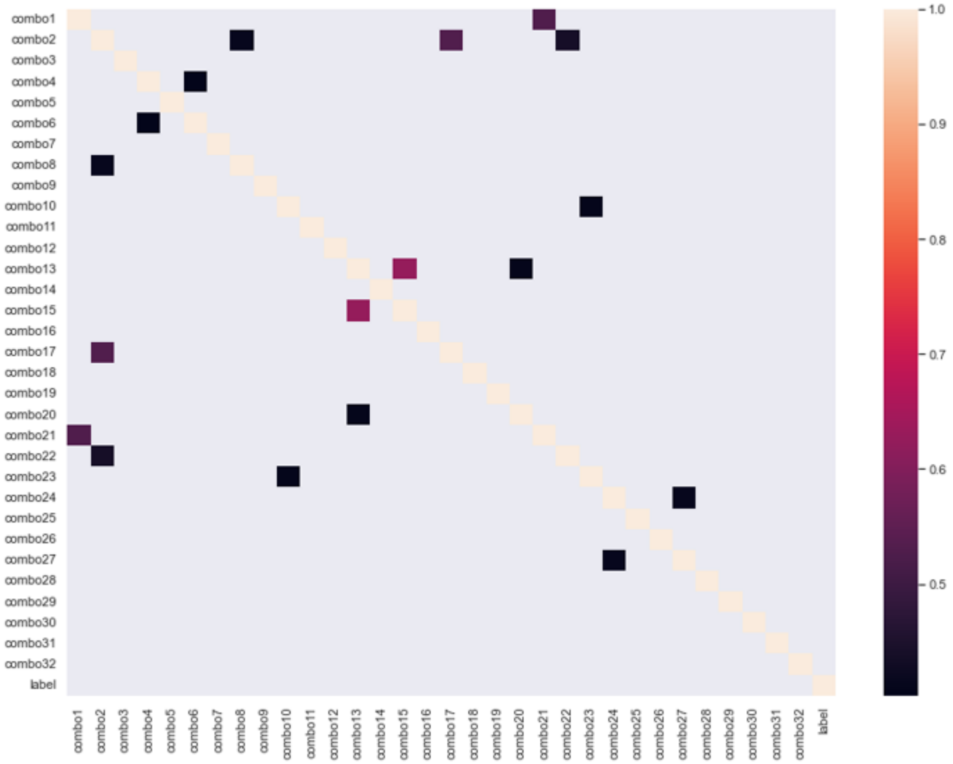

Fig S3. Correlation matrix of 32 embeddings from Combo Model. We filtered the correlation matrix by absolute value of correlation coefficients greater than 0.3 (A) and 0.4 (B) to come up with Composite Model_{g2} and Composite Model_{g3} respectively. We then bundled combo variables with ones that were correlated to each other (Table 1). The remaining combo variables that were not correlated with any other ones were bundled into “comp3” and “comp7” in grouping strategy 2 and 3 respectively (Table 1).

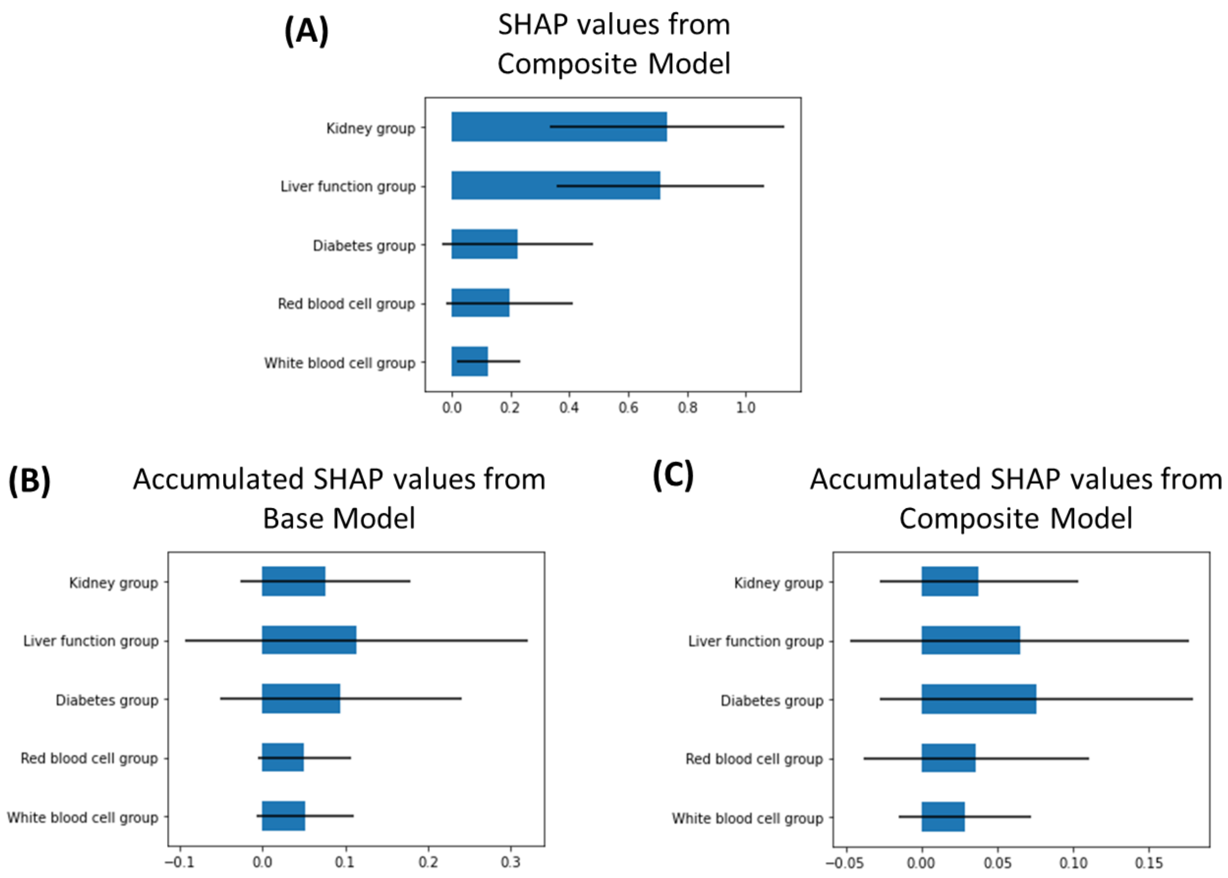

Fig S4. Accumulated feature importance test. (A) SHAP values of 5 composite indices from the Composite Model. Accumulated SHAP values of 5 composite variables from (B) the Base Model and (C) the Composite Model by grouping the SHAP values of 206 individual variables into 5 composite indices.

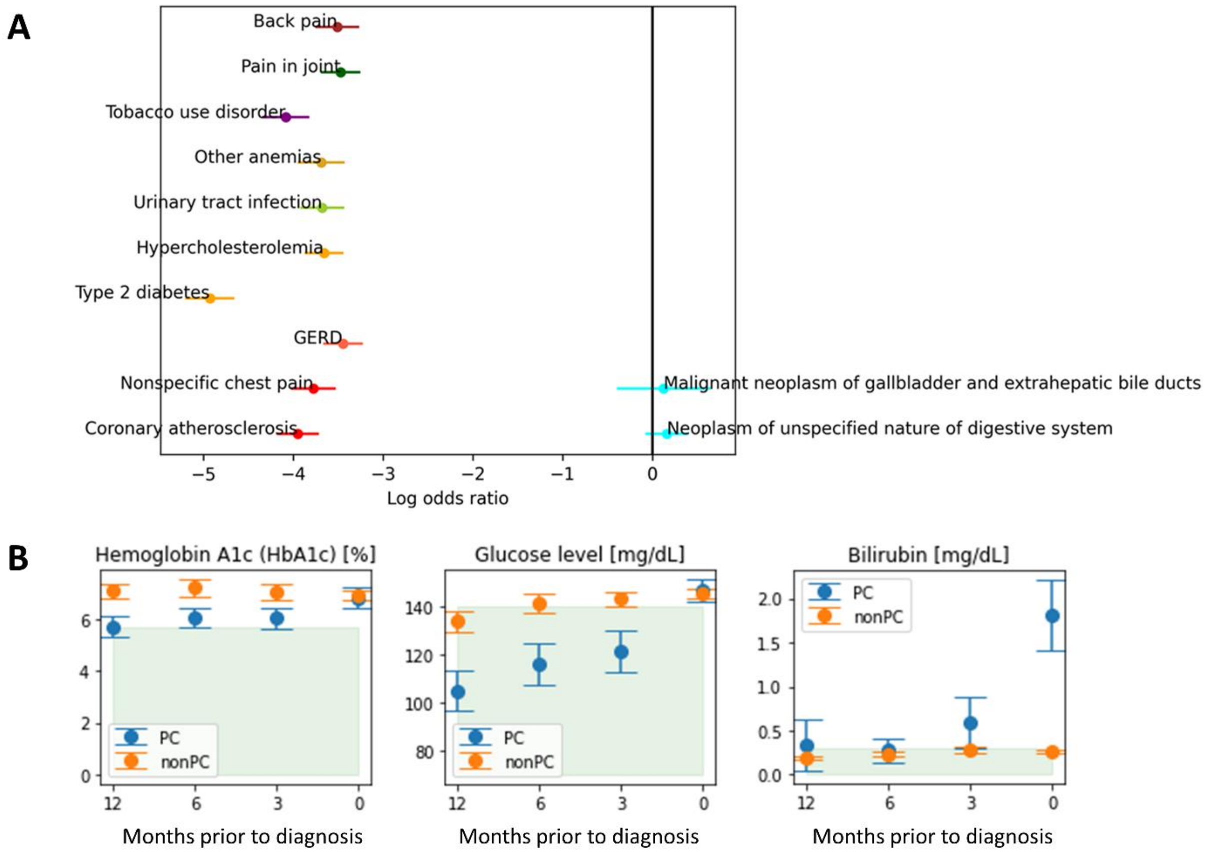

Fig S5. Phenome-wide association study (PheWAS) results. (A) Log Odds Ratio (LOR) plot where top 10 PheCodes resulted in negative LOR and all PheCodes resulted in positive LOR are shown. (B) Temporal changes in time at 0, 3, 6, and 12 months prior to diagnosis. Green shade area indicates normal ranges.

Table S1. Baseline characteristics. This table shows brief baseline characteristics for the final dataset used in the analysis. A full demographics include 7 categories of race, 8 categories of ethnicity, 66 categories of language, and 103 categories of zip codes, which are not shown in this table.

		**PC/nonPC**	
**Total**		**1176 (8%)/14,352 (92%)**	
Risk factors	Smoking	Yes	215 (18%)/2,670 (19%)
		Not documented	961 (82%)/11,682 (81%)
	Obesity	Yes	235 (20%)/2,944 (21%)
		Not documented	941 (80%)/11,408 (79%)
	Diabetes	Yes	880 (75%)/11,098 (77%)
		Not documented	296 (25%)/3,254a (23%)
Demographics	Race	White	543 (46%)/6,284 (44%)
		Asian	36 (3%)/368 (3%)
		African American	144 (12%)/1,882(13%)
		Other Combinations not described	103 (10%)/1,451 (10%)
		Unknown	344 (29%)/4,288 (30%)
	Ethnicity	Caucasian	21 (2%)/280 (2%)
		Hispanic	9 (1%)/68 (1%)
		Not Hispanic	240 (20%)/2,453 (17%)
		African American	124 (11%)/1,517 (10%)
		Unknown	778 (66%)/9,981 (70%)
	Sex	Male	631 (54%)/7,644 (53%)
		Female	545 (46%)/6,708 (47%)
	Zip code	Starts with 0 (MA, NH, ME, VT, CT, NJ)	186 (16%)/1,904 (13%)
		Starts with 1 (NY, PA)	958 (82%)/12,029 (85%)
		Starts with 3 (GA, FL, AL, TN, MS)	20 (2%)/215 (2%)

Language	English	637 (55%)/7,440 (53%)
	Spanish	103 (9%)/1,520 (11%)
	Other	311 (27%)/4,109 (29%)
	Unknown	105 (8%)/1,035 (7%)
Age		73.9 (CI95%=73.2-74.6)/74.5 (CI95%=74.3-74.7)

Table S3. Performance comparison of model results We performed 10 repetitive experiments for each model by randomly splitting the dataset into train set (80%) and test set (20%), and presented mean AUROC and AUPRC with 95% confidence intervals.

**Prediction model**	**Train set**		**Test set**	
	AUROC	AUPRC	AUROC	AUPRC
Base Model	0.873 ± 0.004	0.473 ± 0.010	0.846 ± 0.008	0.410 ± 0.020
Combo Model	0.888 ± 0.005	0.524 ± 0.017	0.855 ± 0.010	0.436 ± 0.022
Composite $Model_{g1}$	0.893 ± 0.004	0.538 ± 0.009	0.858 ± 0.009	0.435 ± 0.033
Composite $Model_{g2}$	0.893 ± 0.005	0.539 ± 0.020	0.859 ± 0.008	0.444 ± 0.025
Composite $Model_{g3}$	0.888 ± 0.006	0.523 ± 0.018	0.854 ± 0.011	0.432 ± 0.029
